# Intraoperative Anaphylaxis to Isosulfan Blue: Recognizing a Rare but Life-Threatening Reaction

**DOI:** 10.7759/cureus.91793

**Published:** 2025-09-07

**Authors:** Brianne Falatovich, Makayla Gologram, D'Arcy Duke

**Affiliations:** 1 General Surgery, Conemaugh Memorial Medical Center, Johnstown, USA; 2 Bariatric Surgery, Conemaugh Memorial Medical Center, Johnstown, USA

**Keywords:** anaphylaxis, isosulfan blue, lymphatics, melanoma, sentinel lymph node biopsy

## Abstract

Isosulfan blue injection (Lymphazurin) is a blue dye injection that is commonly used to map lymphatics in lymphangiography and sentinel lymph node biopsies. A 75-year-old male with a history of hyperlipidemia presented after excision of multiple lesions with plastic surgery, revealing melanoma in situ on the patient’s scalp and superficial spreading melanoma with 4 mm margins on the right posterior axilla. During what was expected to be a routine procedure, anesthesia informed the operating room that the patient had become abruptly hypotensive, but responded to a one-time dose of peripheral vasopressors. The patient's chest and face were noticed to be erythematous and edematous when the patient was turned to begin the wide local excision. Welts and hives were noticed to be originating from the area where isosulfan blue was injected. The patient did fully recover from the incident with no unfortunate sequelae, but given this likely anaphylactic event, the patient should never again be exposed to isosulfan blue without direct physician care. Most reactions to isosulfan blue occur on first exposure and, despite its misnomer, have little to no relation to sulfa allergies. This case report and literature review critique the uses and indications for isosulfan blue, as well as discuss the hazards and prevention of these potentially critical reactions.

## Introduction

Isosulfan blue injection (Lymphazurin) is a commonly used blue dye injection that maps the lymphatic system for the purposes of lymphangiography and sentinel lymph node biopsies (SLNBs) [1]. While commonly used for breast cancer lymph node tracking and biopsy, the use of isosulfan blue is indicated for lymph node involvement by primary or secondary neoplasms. Commonly, isosulfan blue is used in conjunction with radiotracer technetium-99 for greater detection of the desired lymphatic channels, increasing detection rates for breast cancer and melanoma to 99.04% and 97.9%, respectively [2]. As its use has increased, reports of adverse reactions have increased. However, reports of adverse reactions may be limited as isosulfan blue can be difficult to identify as a culprit allergen, and mild reactions may be overlooked. The overall incidence of hypersensitivity reactions in patients is between 0.6% and 2.5%, with reactions ranging from local dermal responses to life-threatening anaphylaxis [3]. Isosulfan blue injection 1% is contraindicated in those individuals with known hypersensitivity to triphenylmethane or related compounds [4]. A history of sulfa allergy is not a known contraindication to use.

Methylene blue is frequently used and has a lower incidence of hypersensitivity than Lymphazurin; however, hypersensitivity can still occur. Methylene blue has a lesser affinity for lymph node uptake, leaving Lymphazurin blue the gold standard for lymph node detection in carefully planned SLNBs. Detection rates for methylene blue and combined radiotracer can be as high as 96%, slightly lower than that of Lymphazurin, but have the benefits of cost-effectiveness and better safety profile regarding allergic reactions [5]. Contraindications for methylene blue include those at risk for serotonin syndrome, patients with G6PD deficiencies, and pregnant women.

In this report, we analyze the clinical scenario of an allergic reaction due to isosulfan blue resulting in anaphylaxis and review the indications for its use as well as alternative methods for lymph node tracing.

## Case presentation

A 75-year-old male with a history of hyperlipidemia presented after excision of multiple lesions with a plastic surgeon. The biopsies revealed melanoma in situ on the patient’s scalp and superficial spreading melanoma with 4 mm margins on the right posterior axilla. The patient was then referred to the general surgery clinic following these excisions. Given the findings on biopsy, as summarized in Table 1, re-excision for appropriate margins and SLNB were considered, and the patient consented to the surgeries. The patient was taken to the operating room for a wide local excision of malignant melanoma on the right posterior axilla with lymphoscintigraphy and SLNB.

**Table 1:** Biopsy results from the right posterior axilla. Biopsy results regarding this melanoma sample proved that it was an invasive disease process. All of the above factors can contribute to the decision to perform a sentinel lymph node biopsy (SNLB). The Clark level and Breslow thickness deemed this biopsy high risk, and further investigation with an SNLB was required.

Malignant melanoma, superficial spreading type (right axilla)	
Clark level	3
Breslow thickness	0.9 mm
Ulceration	Negative
Vascular invasion	Negative
Perineural invasion	Negative
Tumor regression	Positive
Peritumoral lymphocytic response	Brisk
Mitotic activity	Negative
Tumor satellites	Negative
Resection margins	Negative
Nearest peripheral margin	0.4 cm

In the nuclear medicine suite prior to the procedure, the skin surrounding the lesion was injected with technetium-99 sulfur colloid. Lymphoscintigraphy was performed, and the patient was taken to the operating room, where general anesthesia was induced. He was then placed in the supine position with the right arm extended to expose the right axillary region. The skin overlying the previous biopsy site was injected intradermally at the right upper back melanoma with approximately 10 mL of isosulfan blue dye prior to the start of the procedure. A gamma probe was used in the right axilla to identify two transcutaneous lymph nodes. The patient was then prepped and draped in the standard surgical fashion at the right axilla.

Prior to the incision, the lymphoscintigraphy demonstrated two lymph nodes in the axilla. A curvilinear incision was made in the right axilla with a #10 blade scalpel and deepened down through the subcutaneous tissue using electrocautery. Both nodes were located with the gamma probe and blue dye, then submitted for pathology. The axilla was thoroughly inspected visually for blue dye and with palpation. No additional lymph nodes were identified. Given that there were no clinically abnormal nodes and no additional radioactive lymph nodes identified, the superficial spreading melanoma site was excised to 1 cm margins, and the procedure was terminated.

During the SLNB, anesthesia informed the operating room that the patient's blood pressure had dropped, but responded to a one-time dose of peripheral pressors (phenylephrine). The patient's chest and face were noticed to be very erythematous and edematous during this event. When the patient was turned to begin the wide local excision, welts and hives were noticed to be originating from the area where isosulfan blue was injected. The patient did respond to epinephrine for a presumed allergic reaction, and the procedure was carried on as intended.

The patient was successfully extubated and moved to the post-anesthesia care unit with no other complications. Upon awakening from anesthesia, the patient did not recall any known drugs or other allergies. He had never had any previous exposure to Lymphazurin blue. Lymphazurin blue was added to the patient’s allergy list to avoid any future serious interactions.

## Discussion

Isosulfan blue is an aniline dye that is commonly used for SLNB, among other indications [6]. Following injection into parenchyma or subcutaneous tissue, the drug binds to interstitial albumin, which is carried predominantly by lymphatics, allowing the dye to accumulate in lymphatic structures for its identification [6]. Most reactions to isosulfan blue occur on first exposure. Prior studies hypothesize that sensitization may occur through organic exposure to patent blue (parent molecule in isosulfan blue dye) and other structurally related triarylmethane dyes used in textiles, cosmetics, and medications [3,6]. If patients with prior adverse reactions to isosulfan blue require a dye in a future procedure, it is recommended to use methylene blue, which has no structural relation to isosulfan blue or patent blue [6].

The pathophysiology of anaphylaxis to isosulfan blue is not well understood. Anaphylaxis is classified as either immunoglobulin E (IgE)-mediated or non-IgE-mediated. While the exact mechanism of anaphylaxis is not identified in this case, postulated mechanisms include IgE antibody development against foreign material, antigen-mediated mast cell degranulation, direct drug stimulation of mast cells, and idiopathic anaphylaxis [1]. The adverse reactions to isosulfan blue have been previously divided into three grades [7,8]. Grade 1 being the mildest reaction, involving urticaria, pruritus, and occasionally a rash with or without hives. Grade 2 involves transient hypotension not requiring the use of vasopressors. Grade 3 is hypotension requiring vasopressors. The case presented is representative of a grade 3 reaction, which emphasizes the importance of recognition of adverse reactions.

Identifying a causative agent of an intraoperative anaphylactic reaction may be a challenge due to multiple pharmacological reactions at play during a given procedure. Severe reactions to isosulfan blue can increase hospital length of stay, as well as impose financial and emotional stress on a patient due to intensive care needs. This case highlights the importance of identifying isosulfan blue as a causative agent of anaphylaxis, so that prompt intervention can be taken and intensive care may be avoided. Correctly identifying the offending agent is essential for documentation in the electronic health record for future avoidance.

Further studies may suggest the use of pre-procedure skin testing and consideration of preemptively dosing with steroids or antihistamines to avoid anaphylaxis intraoperatively. These interventions should be addressed on a risk versus benefit basis, considering additional healthcare costs and the lack of literature support at this time.

## Conclusions

Isosulfan blue (Lymphazurin) is a commonly used aniline dye injection that is widely used for lymphangiography and SLNBs. While commonly used in surgical practice, adverse reactions ranging from local dermal responses to life-threatening anaphylaxis can occur. Although anaphylaxis is uncommon, surgeons and operating room personnel should be aware of the signs of adverse events so that prompt intervention can be taken. Additionally, open communication with operating room staff is vital when administering medications, so that preparations can be made for possible adverse reactions. This case report highlights that identifying isosulfan blue as a causative agent of anaphylaxis is crucial for patient safety and improving patient outcomes. Physicians should not only understand the indication of its use, but also the appropriate alternatives for such reactions, which can also prove to provide patients with the correct standard of care for their disease process, whether that be melanoma, breast cancer, or one of the many other uses for this effective injection.
